# Association between Attention and Heart Rate Fluctuations in Pathological Worriers

**DOI:** 10.3389/fnhum.2016.00648

**Published:** 2016-12-27

**Authors:** Simone Gazzellini, Maria Dettori, Francesca Amadori, Barbara Paoli, Antonio Napolitano, Francesco Mancini, Cristina Ottaviani

**Affiliations:** ^1^Bambino Gesù Children’s HospitalRome, Italy; ^2^Scuola di Psicoterapia Cognitiva S.r.l.Rome, Italy; ^3^IRCCS Santa Lucia FoundationRome, Italy; ^4^Department of Psychology, Sapienza University of RomeRome, Italy

**Keywords:** worry, heart rate variability, reaction times, sustained attention, time-frequency analysis

## Abstract

Recent data suggests that several psychopathological conditions are associated with alterations in the variability of behavioral and physiological responses. Pathological worry, defined as the cognitive representation of a potential threat, has been associated with reduced variability of heart beat oscillations (i.e., decreased heart rate variability; HRV) and lapses of attention indexed by reaction times (RTs). Clinical populations with attention deficit show RTs oscillation around 0.05 and 0.01 Hz when performing a sustained attention task. We tested the hypothesis that people who are prone to worry do it in a predictable oscillating pattern revealed through recurrent lapses in attention and concomitant oscillating HRV. Sixty healthy young adults (50% women) were recruited: 30 exceeded the clinical cut-off on the Penn State Worry Questionnaire (PSWQ; High-Worry, HW); the remaining 30 constituted the Low-Worry (LW) group. After a diagnostic assessment, participants performed two 15-min sustained attention tasks, interspersed by a standardized worry-induction procedure. RTs, HRV and moods were assessed. The analyses of the frequency spectrum showed that the HW group presents a significant higher and constant peak of RTs oscillation around 0.01 Hz (period 100 s) after the induction of worry, in comparison with their baseline and with the LW group that was not responsive to the induction procedure. Physiologically, the induction significantly reduced high-frequency HRV and such reduction was associated with levels of self-reported worry. Results are coherent with the oscillatory nature of the default mode network (DMN) and further confirm an association between cognitive rigidity and autonomic nervous system inflexibility.

## Introduction

Excessive worry is a core symptom of generalized anxiety disorder (GAD; DSM-V) and has been conceptualized as a chain of thoughts and images, negatively affect-laden and relatively uncontrollable, containing the possibility of one or more negative outcomes and closely related to the fear process (Borkovec et al., 1983). However, worry is definitely not restricted to psychopathology, in fact it can be extremely pervasive also in people who do not meet a former diagnosis of GAD (Ruscio et al., 2001). In this context, the Penn State Worry Questionnaire (PSWQ; Meyer et al., 1990) is a reliable screening measure for pathological worry in GAD and in non-pathological individuals (Beck et al., 1995).

Pathological worry has been associated with several dysfunctional consequences both at a somatic level (chronic physiological activation; Brosschot et al., 2006) and at a cognitive level (impoverished sustained attention; Rapee, 1993). For instance, when given instructions to actively worry about a personally relevant topic, individuals with high levels of self-reported worry report more negative thought intrusions during an attention focusing task compared with those with low levels of self-reported worry (Borkovec et al., 1983). Consistently, Hayes et al. (2008) have shown that—compared with thinking about other topics—worry depletes the ability to exert attentional control, particularly in pathological worriers. Moreover, Fox et al. (2015) showed that dispositional differences in trait propensity to worry are related to difficulties in ignoring irrelevant material with a significant correlation between the degree of deficit in attentional control and the degree of difficulty in suppressing negative thought intrusions. Ottaviani et al. (2013, 2016b) confirmed that a worry induction is associated with a slowing down in reaction times (RTs) during a sustained attention task, further revealing an association between such attentional/cognitive rigidity and autonomic inflexibility, indexed by reduced heart rate variability (HRV). This association has been demonstrated using both subjective measures of cognitive rigidity (Ottaviani et al., 2013) and neural markers of attentional capacity (Ottaviani et al., 2016b). As to the latter, results indicated an association between difficulties in inhibiting worrisome thoughts (both subjectively reported and indexed by RTs slowing down) and impaired deactivation of areas belonging to the so-called default mode network (DMN; Ottaviani et al., 2016b).

The DMN activates during resting states, i.e., when the individual is awake but not actively engaged and the mind is free to wander (Northoff and Bermpohl, 2004; Doucet et al., 2011, 2012). Previous electroencephalography (EEG)- and functional magnetic resonance imaging (fMRI)-based studies identified the low frequency range (0.01–0.1 Hz) as the range within which the DMN pulses (Buzsáki and Draguhn, 2004; De Luca et al., 2006; Balduzzi et al., 2008; Helps et al., 2008; Knyazev et al., 2011; Doucet et al., 2012). According to the Default Mode Interference Hypothesis (Sonuga-Barke and Castellanos, 2007), DMN deactivation would never be complete in the presence of attention deficits; instead, the DMN would intrude during the execution of active tasks, causing lapses in attention (Weissman et al., 2006).

Rather than being random, the attentional falls follow a periodic pattern and the frequency of such lapses in attention is likely to follow the intrinsic frequency of DMN activation. For example, recent studies using time-frequency analysis (e.g., fast Fourier or wavelet transform) in children with Attention-Deficit/Hyperactivity Disorder (ADHD) reported peculiar RTs oscillations around a peak of 0.05 Hz, indicating lapses in attention occurring about every 20 s (Castellanos et al., 2005). Besides, this oscillation pattern proved to be a good predictor of ADHD diagnosis (Di Martino et al., 2008). Subsequent studies on ADHD mostly employed flanker tasks or sustained attention tasks and consistently found significant oscillation peaks in the very low frequency range (0.027–0.073 Hz; Johnson et al., 2007a,b; Di Martino et al., 2008; Adamo et al., 2014).

The Default Mode Interference Hypothesis has also been used as a plausible explanation for the sustained attention deficit of young patients with frontal lesions after traumatic brain injury (Gazzellini et al., 2016). Gazzellini et al. (2016) applied continuous wavelet transform (CWT) to RTs and theta/beta (qEEG) time series. In order to enhance sensitivity in the low-frequency range, attentional tasks duration was kept longer (up to 15–19 min) compared to that used in previous studies. Results showed significant high-power oscillations around 0.01 Hz in traumatic brain injury patients’ performance but not in that of controls for both RTs and theta/beta time series. Results from this and the above-mentioned ADHD studies seem to suggest that very low-frequency oscillation of RTs is a transdiagnostic feature linked to sustained attention deficits irrespective of the underlying specific pathological condition. Indeed, a general increase in RTs variability during attention demanding tasks has been considered as a behavioral biomarker of several psychopathological and neurological conditions (e.g., in bipolar disorder, schizophrenia, ADHD, traumatic brain injury, neurodegenerative pathologies), even in the absence of differences with healthy controls in terms of mean RTs (for a review, see MacDonald et al., 2006).

The main aim of the present study is to determine whether persons who are highly prone to engage in worrisome thoughts do it in a predictable oscillating pattern revealed through increased RTs variability, recurrent lapses in attention, and concomitant oscillating Heart Rate (HR). Such a pattern would be consistent with the hypothesis of a recurrent and intrusive DMN activation during goal-oriented activity (externally directed cognition; Dixon et al., 2014) and the related failure in deactivating such midline structures activity. Given the previously reported association between autonomic and cognitive rigidity, we hypothesize that High-Worry (HW) individuals would show a distinctive pattern of low-frequency spectral power (around 0.01–0.05 Hz) in both HRV and RTs time series, revealing lapses in attention during the execution of a sustained attention task. Lastly, we hypothesized these oscillatory patterns to be associated with state and trait psychological characteristics of the individual.

## Materials and Methods

### Participants

Participants were recruited by the use of flyers and participation in previous studies. The sample was composed of 60 subjects (31 women, 29 men; mean age = 30.4 (6.9) years). The cut-off score for pathological worry on the PSWQ (Meyer et al., 1990) was used to pre-assess eligibility of both pathological worriers (≥54; *n* = 30) and controls (<54; *n* = 30). This cut-off has been recommended for optimal sensitivity and specificity in selected samples (Salzer et al., 2009). Exclusionary criteria were: being younger than 18, a diagnosis of psychiatric disorder, a diagnosis of heart disease or any other serious illness, use of drugs/medications that might affect HR and HRV, obesity (body mass index (BMI) > 32 kg/m^2^), menopause, pregnancy or childbirth within the last 12 months. Participants were compensated (€15) for their time. The protocol was approved by the Bioethical Committee of S. Lucia Foundation, Rome, Italy.

### Procedure

After eligibility assessment, participants came to the lab, read and signed the informed consent form, and filled out a series of questionnaires. Then, electrocardiogram electrodes were attached to the subject and participants performed a sustained attention task for 15 min. After the task, participants underwent a verbal induction procedure designed to engender perseverative cognition (i.e., rumination and worry; 5 min). Then, participants performed again the same sustained attention task for 15 min. Before and after performing each task, participants rated their thoughts and moods over the preceding period using visual analog scales. Psychophysiological data were recorded throughout the session.

### Questionnaires

Participants completed a series of socio-demographic and lifestyle (nicotine, alcohol and caffeine consumption, physical exercise) questions and questionnaires to measure levels of: (a) trait rumination (Ruminative Response Scale, RRS; Nolen-Hoeksema and Morrow, 1991); (b) state and trait anxiety (State-Trait Anxiety Inventory, STAI-X2; Spielberger et al., 1970); and (c) depression (Beck Depression Inventory, BDI-II; Beck et al., 1996).

The PSWQ is a 16-item self-report questionnaire commonly used to assess pathological worry in both clinical and non-clinical populations. It has been shown to have good internal consistency with samples consisting of older adults with GAD (Beck et al., 1995), community subjects (Brown et al., 1992) and undergraduates (Meyer et al., 1990). The PSWQ is positively correlated with other self-report measures of worry (e.g., Davey, 1993; Beck et al., 1995; Van Rijsoort et al., 1999). The internal reliability (Cronbach’s alpha = 0.92) and psychometric properties of the Italian version of the PSWQ have been demonstrated to be satisfactory (Meloni and Gana, 2001).

The RRS assesses depressive rumination measured by how often people engage in responses to depressed mood that are self-focused (I think “Why do I react this way?”), symptom-focused (I think about how hard it is to concentrate), and focused on the possible consequences and causes of one’s mood (I think “I won’t be able to do my job if I don’t snap out of this”).

The STAI consists of two 20-item self-report measures to assess state and trait levels of anxiety. Respondents indicate how they feel right now (state version) or how they generally feel (trait version) using four-point Likert scales.

The BDI-II requires participants to respond how each of 21 statements relates to the way they have felt for the past 2 weeks. This instrument is intended to assess the existence and severity of symptoms of depression as listed in the American Psychiatric Association (1994).

### Attentional Task

As a sustained attention to response task, we used a modified version of the Continuous Performance Test (Conners, 2000), adapted for the aims of the present study. Participants were required to respond to all the letters (go condition), with the exception of the consonant “Z” (no-go condition) by pressing the space bar as quickly and accurate as possible. Black letters (size 1 cm × 1.4 cm) appeared on a white background at the center of the screen. In order to present the stimuli foveally, they were included in a 2° horizontal visual angle; the distance between participants and the monitor was 80 cm. The task comprised 528 randomly presented stimuli, 48 no-go trials and 480 go trials, without any block division. Task duration was 15 min. The inter stimulus interval was 1700 ms.

The stimuli appeared on a video display unit controlled by an IBM Personal Computer. The software E-Prime version 2.0 (Schneider et al., 2002) was used for visual presentation of the stimuli and data collection. The timing accuracy of the software is ± 0.5 ms.

### Induction

“Next I would like you to recall an episode that happened in the past year that made you feel sad, anxious, or stressed, or something that may happen in the future that worries you. Then, I would like you to think about this episode in detail, for example about its possible causes, consequences, and your feelings about it. Please take as much time as you need to recall the episode and tell me about it whenever you are ready”.

The experimenter recorded: (1) the topic selected by each participant during the induction; (2) its temporality (past or future); and (3) temporal distance (how long in the past/how far in the future).

### Visual Analog Scales

At the beginning and at the end of the sustained attention task, participants were asked to rate their current levels of feeling sad, calm and worried on separate visual analog 100-point scales. For each mood, change scores (task value minus initial baseline value) were computed by subtracting the initial baseline from task values.

### Psychophysiological Assessment and Pre-Processing

HR was recorded as beat-to-beat intervals in ms with the Bodyguard 2 (Firstbeat) HR monitor that has been extensively used for HR recording and analysis (e.g., Ottaviani et al., 2015). Frequency-domain measures of HRV were obtained using Kubios Analysis Software (Tarvainen et al., 2014): low-frequency HRV (LF-HRV), high-frequency HRV (HF-HRV), and LF/HF-HRV. According to the Task Force of the European Society of Cardiology and the North American Society of Pacing and Electrophysiology (1996), the HF-HRV (0.15–0.4 Hz) reflects parasympathetic activity, and the LF-HRV (0.04–0.15 Hz) is proportional to sympathetic activity but influenced by parasympathetic tone. The interpretation of LF-HRV as primarily an index of sympathetic tone has been commonly derived by the calculation of the ratio of LF/HF-HRV. The time series of inter-beat intervals for each participant in the two conditions (before and after induction) were extrapolated from the device. Inter-beat intervals that corresponded to a HR below 30 or above 200 were excluded, as well as any interval resulting in an increase or drop in HR >30% between successive intervals (2.1% of the data). Deleted data were linearly interpolated.

### Reaction Times Pre-Processing

RTs under the physiological threshold of 100 ms were considered as anticipations and removed from the distribution. Error variability in the sustained attention task was regressed out by subtracting the corresponding trial type mean from each value. The unstandardized regression residuals represent the portion of each RT score that is independent of response type and correctness (procedure already applied by Helps et al., 2011; Gazzellini et al., 2016). No-go trials, missing data (non-responses to go trials), and anticipations were discarded and linearly interpolated to maintain the temporal structure of the time series.

### Continuous Wavelet Transform

CWT is a powerful tool allowing to decompose a continuous-time function into wavelet functions and therefore it is very useful to retrieve the frequency content of the function. The principal difference between the Fourier Transform and the wavelet is that the wavelets are localized both in time and frequency whereas the standard Fourier transform is localized only in frequency. Consequently, the CWT possesses the ability to construct a time-frequency representation of a signal that offers very good time and frequency localization. The CWT with Morlet wavelets with half length of Morlet analyzing wavelet at the coarsest scale equal to 20 was applied to each subject’s normalized time series (RTs and inter-beat intervals), obtaining the spectral density of the signal varying over time: scalogram. The scalogram was averaged over the whole task interval to attain the average spectral power per frequency. The maximum powers in each pre-determined range was automatically computed and these values were taken as the dependent variables in the Analysis of Variances (ANOVAs) and Fisher’s LSD *post hoc* tests. We adopted the frequency ranges selected by Penttonen and Buzsáki (2003), who argued that a natural logarithmic relationship links brain oscillators from the ultraslow to ultrafast frequencies: Slow-6 (0.0052 Hz–0.010 Hz, centered at 0.006 Hz [period 101–192 s]), Slow-5 (0.010–0.027 Hz, centered at 0.016 Hz [37–101 s]), Slow-4 (0.027–0.073 Hz, centered at 0.044 Hz [14–37 s]), and Slow-3 (0.073–0.17 Hz, centered at 0.12 Hz [6–14 s]). This frequency classification has been previously adopted in dedicated studies (Di Martino et al., 2008; Helps et al., 2008; Gazzellini et al., 2016).

### Statistical Analyses

Data are expressed as means standard deviation (SD). *P*-values ≤ 0.05 were considered as significant. Laboratory data processing and analyses were performed using STATISTICA (Statsoft, Inc.). Kolmogorov-Smirnov test was used to test the normality of all variables. The distribution for LF-HRV, HF-HRV and LF/HF-HRV was non-normal; therefore these variables were log transformed (ln).

First, pre-existing group and gender differences were analyzed by *t*-tests.

Second, a series of 2 (Group: High Worriers vs. Low Worriers) × 2 (Induction: Before vs. After) ANOVAs were performed on: (a) HR, ln (LF-HRV), ln (HF-HRV) and ln (LF/HF-HRV); (b) levels of being sad, calm and worried (visual-analog scales); (c) mean, SD and coefficient of variability (CV; SD/Mean) of RTs; and (d) and percentage of errors. CV has the merit to be a measure of RT variability independent of differences in mean RT (Allan Cheyne et al., 2009) and has been used in previous studies on mind wandering and behavioral variability (e.g., Baird et al., 2014).

Third, mixed three-way ANOVAs (Group × Range × Induction: 2 × 4 × 2) with Group as between-subject factor and Range (Slow 6, 5, 4, 3) and Induction as within-subject factors were carried out on RTs and inter-beat intervals series. CWT spectral power peak served as the dependent variable.

Fisher’s LSD *post hoc* tests were executed in case of significant main effects.

Finally, correlational analyses were performed between physiological and attentional responses to the induction and state and trait psychological characteristics of the sample.

## Results

Table 1 shows pre-existing (baseline) group differences for the main variables of the study. The HW had lower BMI compared to Low-Worry (LW) participants (*t* = 1.98, *p* = 0.05). In addition to the PSWQ (*t* = 11.73, *p* < 0.0001), pathological worriers had higher levels of: (a) trait (*t* = −3.16; *p* = 0.002) and state anxiety (*t* = 3.16; *p* = 0.003); (b) depression (*t* = 3.74; *p* < 0.0001); and (c) trait rumination (*t* = 4.99; *p* < 0.0001).

**Table 1:** **Group differences in socio-demographic, personality and baseline mood variables**.

	High-Worry (*n* = 30)	Low-Worry (*n* = 30)	*p*
Gender	19 F; 11 M	12 F; 18 M	0.07
Age (years)	29.6 (7.3)	31.2 (6.5)	0.37
BMI (Kg/m^2^)	21.8 (2.2)	23.3 (3.6)	0.05
Smoking	19 N, 11 Y	16 N, 14 Y	0.43
Alcohol consumption	6 N, 24 Y	3 N, 27 Y	0.28
Caffeine consumption	3 N, 27 Y	1 N, 29 Y	0.30
Exercise	8 N, 22 Y	9 N, 21 Y	0.61
BDI	11.5 (7.9)	5.6 (3.4)	<0.0001
STAI-T	47.3 (7.0)	40.5 (9.4)	0.002
STAI-S	50.9 (8.5)	43.7 (9.1)	0.003
PSWQ	58.5 (5.3)	35.8 (9.7)	<0.0001
RRS	45.6 (13.4)	31.6 (7.4)	<0.0001
Calm	19.9 (21.1)	18.8 (10.5)	0.84
Worried	7.3 (11.3)	3.8 (5.0)	0.13
Sad	4.7 (7.9)	1.7 (3.1)	0.07

*Note. BMI, Body Mass Index; M, Males; F, Females; Y, Yes; N, No*.

No gender differences emerged for any of the examined physiological variables (*p*_s_ > 0.15), therefore gender was not included as a covariate in the subsequent analyses. In light of pre-existing differences between the two groups, BMI was included as a covariate in all the subsequent analyses.

With regard to the Induction, *n* = 1 HW and *n* = 3 LW participants chose to focus on a past episode. All the remaining participants focused on something that worried them in the future. The average temporal distance of the event was 10.8 (18.3) days. In both groups, most participants chose to worry about work-related issues (*n* = 18 in the HW and *n* = 20 in the LW groups), followed by romantic, health and family issues.

Table 2 reports the mean and SDs of the main variables of the study in HW and LW participants before and after the induction, as well as change scores from pre- to post-induction. A significant Group × Induction interaction emerged for HF-HRV (*F*_(1,50)_ = 4.61, *p* < 0.05; $\eta_{p}^{2}$ = 0.10). *Post hoc* comparisons showed that HF significantly decreased from pre- to post-induction in the HW group only (*p* = 0.002). The ANOVAs did not yield significant main effects of Group or Induction. BMI did not play a significant role as a covariate in the model. No significant main effects or interactions emerged for HR, LF-HRV or LF/HF-HRV.

**Table 2:** **Physiological, behavioral and mood variables in High- (HW) and Low-Worriers (LW) during the sustained attention task preceding (Before) and following (After) the induction, and change scores from pre- to post-induction (Δ = After minus Before)**.

	Before induction		After induction		Δ (after-before)	
	HW	LW	HW	LW	HW	LW
HR (bpm)	76.1 ± 9.6	78.8 ± 13.5	77.4 ± 10.1	76.6 ± 11.9	1.39 ± 4.8	−2.3 ± 6.9
HF-HRV	716.2 ± 533.6	555.8 ± 331.3	582.9 ± 459.2	537.2 ± 324.1	−133.3 ± 238.7	−18.6 ± 186.5
LF-HRV	1602.5 ± 921.4	1379.9 ± 1226.2	1568.4 ± 1013.7	1347.6 ± 944.7	−34.1 ± 603.7	−32.4 ± 631.2
LF/HF-HRV	2.9 ± 1.9	2.8 ± 1.6	3.2 ± 2.0	2.9 ± 2.1	2.6 ± 1.2	0.1 ± 1.2
RTs (ms)	373	387	369	372	−4	−15
RTs SD (ms)	84	89	88	86	4	−3
RTs CV (ms)	0.22 ± 0.1	0.23 ± 0.1	0.23 ± 0.04	0.23 ± 0.1	0.01 ± 0.002	0.06 ± 0.03
Errors (%)	2.3 ± 0.3	1.9 ± 0.3	2.7 ± 0.6	3 ± 0.6	0.4 ± 0.3	1.1 ± 0.3
Calm	17.8 ± 19.6	18.7 ± 19.4	19.9 ± 23.0	22.3 ± 24.4	2.1 ± 10.2	3.6 ± 16.1
Worried	11.8 ± 21.8	2.8 ± 4.6	12.7 ± 24.3	3.2 ± 8.2	0.9 ± 5.8	0.4 ± 4.6
Sad	5.4 ± 9.3	1.8 ± 3.6	7.7 ± 16.8	1.1 ± 2.1	2.3 ± 3.4	−0.7 ± 2.7

*Note. HW, High-Worry; LW, Low-Worry; HR, Heart Rate; HRV, Heart Rate Variability; SD, Standard Deviation; CV, Coefficient of Variability*.

A main effect of Group emerged for levels of Worry (*F*_(1,54)_ = 3.91, *p* < 0.05; $\eta_{p}^{2}$ = 0.08) and Sad (*F*_(1,54)_ = 3.99, *p* < 0.05; $\eta_{p}^{2}$ = 0.08) with the HW group having higher levels of self-rated worry and sadness compared to the LW group, irrespective of the Induction. No other significant effect emerged for scores on the visual-analog scales.

A significant main effect of Induction emerged from the ANOVA on average RTs (*F*_(1,58)_ = 6, *p* < 0.05; $\eta_{p}^{2}$ = 0.09). LSD *post hoc* comparisons revealed that mean RTs significantly decreased in the LW group from pre- to post-induction (*p* < 0.01); such an effect was not present in the HW group. Interestingly, a significant pre- to post-induction increase in CV was found in the HW group (*p* < 0.05).

**Figure 1:**
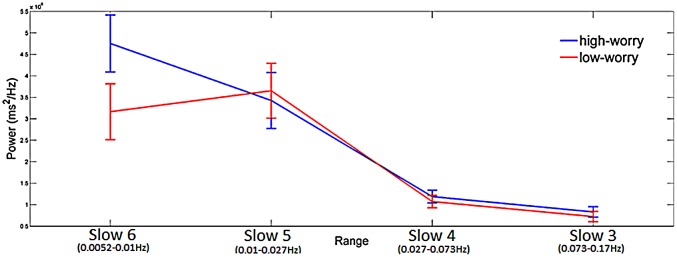
**Significant Range × Group interaction emerging from the Analysis of Variance (ANOVA) having spectral power mean value of inter-beat interval as the dependent variable.** Blue line is for High-Worry (HW) and red line is for Low-Worry (LW) participants. The four ranges (Slow 6, 5, 4 and 3) are reported on the *X*-axis. Mean maximum peak value (power ms^2^/Hz) is reported on the *Y*-axis. *Note*. Vertical bars denote 0.95 confidence intervals.

The group averaged CWT was performed on inter-beat intervals time series acquired during task performance and returned the maximum peak in each of the four frequency ranges. The mixed three-way ANOVA reported a significant main factor of Range (*F*_(3,159)_ = 43.4, *p* < 0.001; $\eta_{p}^{2}$ = 0.45) and a significant Range × Group interaction (*F*_(3,159)_ = 2.7, *p* < 0.05; $\eta_{p}^{2}$ = 0.05; see Figure 1). *Post hoc* analyses on the main factor Range revealed the following differences: Slow 6 = 5 > 4 = 3. *Post hoc* comparisons on the Range × Group interaction showed a significant higher spectral power mean value in the HW group compared to the LW group in Slow 6 (0.0052 Hz–0.010 Hz).

**Figure 2:**
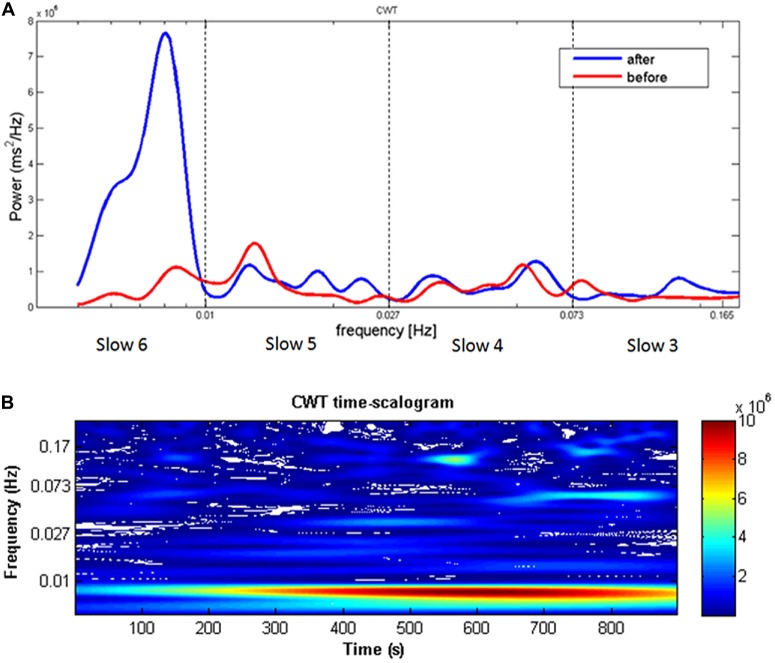
**(A)** Continuous wavelet transform (CWT) reaction time (RT) spectrogram of a single representative HW participant showing a clear peak around 0.01 Hz when comparing before and after the worry induction procedure. The dotted lines represent the boundaries of the four frequency ranges (Slow 6, 5, 4, and 3). Frequency (Hz) is reported on the *X*-axis and spectral power (ms^2^/Hz) on the *Y*-axis. **(B)** Distribution of the power spectrum of RTs collected in the after condition along the time dimension. Warm colors represent higher power values. The signal at 0.01 Hz increases in power after the first 100 s and remains significantly higher with respect to the other frequencies for the entire task duration.

The time series of RTs were also subjected to CWT for the time-frequency power spectrum analysis. Figure 2 depicts the power spectrum of a representative HW participant before and after the induction, showing a clear peak around 0.01 Hz during performance at the second sustained attention task. As far as the time dimension is concerned, the signal at 0.01 Hz increases in power after the first 100 s and remains significantly higher with respect to the other frequencies for the entire task duration (Figure 2B). On the contrary, no evident peaks emerged in LW participants. As a consequence of differences in participants’ spectrograms (inter-subject variability), the group-averaged spectrogram may be affected by single peaks at slightly different frequencies, and therefore may not be as sharp and evident as those of the single subjects.

**Figure 3:**
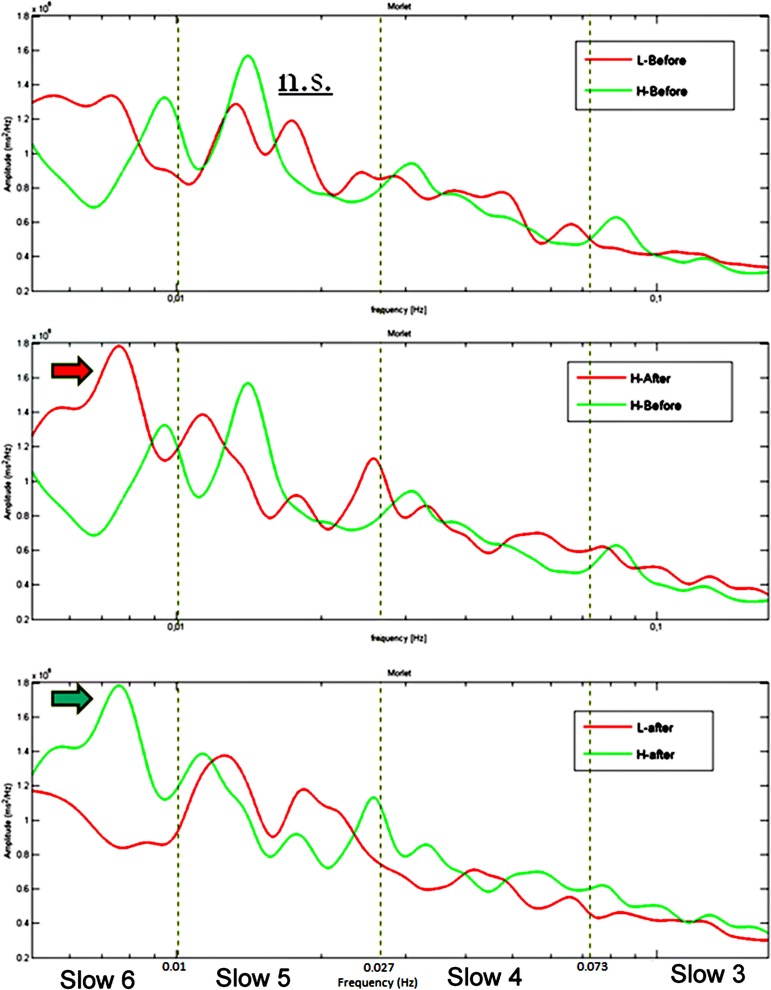
**Group average CWT on RTs time series showing no difference between the two groups at baseline (upper panel), a significant greater peak in Slow 6 (0.0052–0.010 Hz) after the induction compared to before the induction in the HW group (middle panel) and a significant greater peak in Slow 6 in the HW compared to the LW group after the induction (lower panel).** The dotted lines represent the boundaries of the four frequency ranges. Frequency (Hz) is reported on the *X*-axis and spectral power (ms^2^/Hz) on the *Y*-axis.

Group averaged CWT on RTs series acquired during performances at the sustained attention task returned higher spectral powers at VLF for the HW group: (a) compared to those of the LW group; (b) after the induction compared to pre-induction. As shown in Figure 3, a clear power peak around 0.01 Hz is present in the HW group’s spectrogram after the induction but not in the LW group or before the induction.

**Figure 4:**
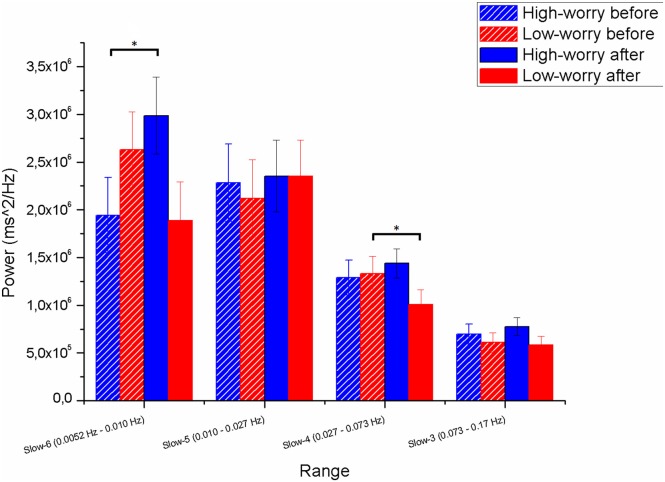
**Means and standard deviations (SD) from the Group × Range × Induction interaction on the dependent variable maximum peak value (power ms^2^/Hz) in the range, on the *Y*-axis.** The four ranges (Slow 6, 5, 4, and 3) and the four conditions (Group × Induction) are reported on the *X*-axis. The horizontal bars point out statistical differences at the LSD *post hoc* test. *Note*. *p < 0.05. Session 1 = before the induction; Session 2 = after the induction. Vertical bars denote 0.95 confidence intervals.

The mixed three-way ANOVA (Group × Range × Induction) on peak power as dependent variable yielded a main effect of Range (*F*_(3,174)_ = 49.7, *p* < 0.001; $\eta_{p}^{2}$ = 0.46), whereas the other main effects did not reach significance. Two significant interactions emerged: Group × Induction (*F*_(3,58)_ = 4, *p* < 0.05; $\eta_{p}^{2}$ = 0.06) and Group × Range × Induction (*F*_(3,174)_ = 4.3, *p* < 0.01; $\eta_{p}^{2}$ = 0.07). As depicted in Figure 4, significant interactions emerged between Group and Induction in Slow 6 and 4 but not in Slow 5 and 3. The interaction in Slow 6 is of particular interest: *post hoc* analysis revealed that whereas HW participant significantly increased their power peaks in Slow 6 after the worry induction (*p* < 0.05), the LW group showed an opposite and marginally significant trend, decreasing power peak values in Slow 6 after the induction (*p* = 0.07). The significant Group × Induction interaction in Slow 4 was due to a significant power decrease in the LW group after the induction (*p* < 0.05).

Correlation analyses (*p*s < 0.05) showed that HRV reactivity to the induction was negatively associated with changes in state worry (*r* = −0.70) from pre- to post-induction (change scores). RT power in Slow 6 before induction was positively correlated with HR (*r* = 0.37) and inversely correlated with LF-HRV (*r* = −0.31) and HF-HRV (*r* = −0.29). The RT power in Slow 6 after the induction was positively correlated with state anxiety (*r* = 0.27) and increases in levels of sadness from pre- to post-induction (*r* = 0.26). HR power in Slow 6 in the HW group after—but not before—the induction was positively correlated with BDI score (*r* = 0.45).

## Discussion

The ability to adjust bodily reactions in response to a changing environment and effectively ignore irrelevant information is crucial for adaptive behavior. The aim of the present study was to investigate the association between such autonomic and cognitive flexibility during worry, by analyzing RTs and HR fluctuations in pathological worriers and controls. To do so, we asked HW and LW participants to perform two sustained attention tasks interspersed by a worry induction and we applied CWT to RTs and inter-beat intervals.

First, previously reported differences between HW and LW in terms of psychopathological characteristics were replicated. Pathological worriers without GAD had higher levels of trait and state anxiety and depression (Ruscio, 2002; Hirsch and Mathews, 2012; Ottaviani et al., 2014). Interestingly, high-worriers were also characterized by higher levels of trait rumination, confirming the usefulness of merging rumination and worry under a unique transdiagnostic construct (e.g., perseverative cognition, repetitive negative thinking; McEvoy et al., 2013).

Irrespective of the induction, pathological worriers reported higher levels of state worry and sadness compared to controls. The fact that levels of worry did not significantly increase after the induction in participants who are prone to engage in this cognitive process may appear surprising. However, this is not an unusual finding and—like in previous studies conducted in patients with GAD—it is simply due to the already higher baseline levels of state worry in these populations (e.g., Makovac et al., 2015).

Participants in the present study chose work-related issues as the most common topic to worry about. This is understandable if we consider that our sample was mostly composed by university students ready to enter the job market at times of economic crisis. Work-related worry and rumination have been previously associated with dysfunctional consequences at a physiological level, such as flattened cortisol awakening response (Cropley et al., 2015).

In the present study, the induction of work-related and—to a lesser extent—other types of worries had the consequence to decrease vagally-mediated HRV in pathological worriers only, although decreases in HRV were strongly associated with increases in levels of self-reported state worry in the entire sample. Present results are in line with Ottaviani et al. (2014), in which physiological responses to unpredictable bursts of loud white noise were characterized by lower vagally-mediated HRV in HW but not in LW. Indeed, reduced HRV has been proposed as a biomarker of worry, irrespective of the presence of a specific anxiety disorder (Chalmers et al., 2016). The absence of a HRV decrease during worry in controls seems to be in contradiction with data from a recent meta-analysis showing that vagal withdrawal is a signature of worry in *non-pathological* subjects (Ottaviani et al., 2016a). Such an apparent incongruity may be explained by the existence of pathological worry in the absence of a frank psychiatric diagnosis (Ruscio, 2002). For this reason, high-worriers have likely been included in the healthy population examined in the above-mentioned meta-analysis (Ottaviani et al., 2016a). It is imperative that future studies examining worry in healthy population include a measure of dispositional worry and test for potential differences between HW and LW.

The two groups in our study were not only different in terms of autonomic response but also in attentional performance. Whereas RTs decreased in non-worriers during second performance in the sustained attention task, likely indicating a learning effect, this was not the case for pathological worriers. Moreover, only pathological worriers increased their coefficient of RT variability after the induction, whereas their mean RTs did not change. This finding suggests that behavioral variability—instead of average velocity—might be assumed as a biomarker for pathological states, as already documented for other psychopathological and neurological conditions (Castellanos et al., 2005; MacDonald et al., 2006; Gazzellini et al., 2016). The lack of performance improvement in high-worriers and the concomitant increase in the CV possibly signal the presence of intrusive thoughts as suggested by previous studies linking deficits in attentional control to greater difficulty in controlling negative thought intrusions (Fox et al., 2015).

Building on these results, the present study had the aim to provide further evidence that, instead of being random, negative thought intrusion might follows periodic physiological oscillations. The time frequency analysis (CWT) showed that both behavioral and cardiac-autonomic indices oscillate in a very low frequency range (0.005 Hz–0.01 Hz; period 101–192 s) in pathological worriers with a peak value just before 0.01 Hz. In particular, data on RT series in the HW but not in the LW group showed a clear increase of power in the 0.005–0.01 Hz range after the worry induction procedure, suggesting an increase of behavioral variability around 0.01 Hz and therefore possibly the increase of negative thought intrusions at a regular oscillation around 100 s (see Figures 2, 3). Therefore, not only pathological worriers were characterized by increased RTs variability, as documented by the traditional RTs analyses, but such behavioral variability also oscillated within the specific frequency range of 0.005–0.01 Hz. On the contrary, the LW group showed power decrease in the Slow 6 and 4, probably due to a learning effect, which was absent in worriers and is consistent with the already described learning effect indexed by mean RTs.

Time frequency analysis on inter-beat intervals also revealed significant higher powers in the same range (0.005–0.01 Hz) but, in this case, this was already present in worriers at the baseline evaluation and hence did not show any further increase after the worry induction. Taken together, present data may suggest that inter-beat intervals oscillations reflect a “trait” characteristic of high worriers, whereas RT oscillations might be a more sensitive index of “state” pathological worry.

The oscillation frequency of RTs and inter-beat intervals in the 0.005–0.01 Hz range found in this study is consistent with previous observation of DMN frequency activation (Buzsáki and Draguhn, 2004; Vanhatalo et al., 2004; De Luca et al., 2006; Balduzzi et al., 2008; Doucet et al., 2012) and frequency of recurrent lapses in attention in frontal brain damaged patients with attention deficits (Gazzellini et al., 2016). In light of present data, it is reasonable to assume that also in pathological worriers a recurrent activation of DMN may cause propensity to lose attention about every 100 s during goal-oriented activity. Interestingly, data revealed that also HR oscillates at the 0.005–0.01 Hz range in pathological worriers linking its variability to regular lapses in attention during task execution. Such frequency range encompasses the very low- (0.0033–0.04 Hz) and low-frequency (0.04–0.15 Hz) components usually adopted for HRV analysis Task Force of the European Society of Cardiology and the North American Society of Pacing and Electrophysiology (1996).

Intriguingly, a negative correlation emerged between RT power in the low frequency range—which seems to be the range within which the DMN pulses—and vagally-mediated HRV. Although indirect, this result adds to the increasing evidence in favor of an association between vagal and DMN activity (Thayer et al., 2012 for a meta-analysis; Jennings et al., 2016). In the context of worry, current data support a recent imaging study in which individuals with high trait perseverative cognition had more difficulties suppressing DMN activity during detection of infrequent targets, and the magnitude of such activity change was predicted by individual differences in HRV (Ottaviani et al., 2016b). Present data are relevant, as they constitute a further proof of the association between the autonomic rigidity and cognitive inflexibility as a signature of perseverative cognition.

A limitation of the present study is that our pathological sub-sample was an “above the cut-off” group, making it difficult to generalize results to psychopathological disorders. Second, we did not collect direct evidence of low-frequency fluctuations in the DMN obtained via fMRI or EEG. Third, the physiological meaning of very low frequencies is disputable, mainly due to the fact that such frequency band is highly affected by algorithms of trend removal. For this reason, the Task Force of the European Society of Cardiology and the North American Society of Pacing and Electrophysiology (1996) recommend to avoid the interpretation of very low frequencies for short-term recordings of less than 5 min. However, the present study had 15-min recordings and participants were sitting without moving except for pressing the computer space bar, therefore it is unlikely that the reported differences reflect technical artifacts instead of physiological functions. Moreover, the percentage of artifacts was quite low (2.1%), suggesting a reliable electrocardiographic signal. Moreover, previous studies showed higher correspondence between relative power of very low frequency and lower baroreflex sensitivity coupled with lower gain of its efferent component regulating cardiac rhythm, compared to other HRV bands (Davydov et al., 2007). Relative power within the very low frequency band has also been associated with depressive symptoms in children and adolescents (Blood et al., 2015) and predicted changes in depression (with lower very low frequency representing a marker of good prognosis) and treatment outcome in adults with major depression, suggesting its potential role as a biomarker for psychological wellbeing. Other compelling reason to exclude that current results on very low frequency may simply represent an artifact are the following: (a) peaks in this range do not appear randomly but only in the HW group and in the post-induction condition; (b) we have found the same significant differences on RTs, which are free from problematic issues as sweating, movement and electrode drift; and (c) differences in the same frequency range were similarly found in RTs and EEG data by Gazzellini et al. (2016) when comparing frontal patients and healthy controls. Keeping in mind that replications are necessary to clarify this issue, the fact that signals at the 0.0052–0.01 Hz range are typically removed from the analyses and that task duration is usually below 15 min may have hidden frequency peaks in such range in previous studies.

In sum, persons who are highly prone to engage in worrisome thoughts do it in a predictable oscillating pattern revealed through increased RTs variability, recurrent lapses in attention, and concomitant oscillating HR. Pathological worry is associated with detrimental outcomes at a cardiac (decreased HRV), cognitive (increased propensity to lapses in attention) and behavioral levels (increased RTs variability). At a central nervous system level, this association is presumably mediated by the midline cortical structures belonging to the DMN.

## Author Contributions

SG, CO, FM conceived and designed the experiments. MD, FA, BP performed the experiments. SG, CO, AN analyzed the data. AN, MD, FA, BP contributed materials/analysis tool. SG, CO, AN, FM, MD, FA, BP contributed to the writing of the manuscript.

## Conflict of Interest Statement

The authors declare that the research was conducted in the absence of any commercial or financial relationships that could be construed as a potential conflict of interest.

## References

[B2] Adamo N. Di Martino A. Esu L. Petkova E. Johnson K. Kelly S.. (2014). Increased response-time variability across different cognitive tasks in children with ADHD. J. Atten. Disord. 18, 434–446. 10.1177/1087054712439419 22508759

[B15] Allan Cheyne J. Solman G. J. F. Carriere J. S. A. Smilek D. (2009). Anatomy of an error: a bidirectional state model of task engagement/disengagement and attention-related errors. Cognition 111, 98–113. 10.1016/j.cognition.2008.12.009 19215913

[B3] American Psychiatric Association (1994). Diagnostic and Statistical Manual of Mental Disorders: (DSM-IV). 4th Edn. Washington, DC: American Psychiatric Association.

[B4] Baird B. Smallwood J. Lutz A. Schooler J. W. (2014). The decoupled mind: mind-wandering disrupts cortical phase-locking to perceptual events. J. Cogn. Neurosci. 26, 2596–2607. 10.1162/jocn_a_00656 24742189

[B5] Balduzzi D. Riedner B. A. Tononi G. (2008). A BOLD window into brain waves. Proc. Natl. Acad. Sci. U S A 105, 15641–15642. 10.1073/pnas.0808310105 18843102 PMC2572977

[B7] Beck J. G. Stanley M. A. Zebb B. J. (1995). Psychometric properties of the Penn State Worry Questionnaire. J. Clin. Geropsychology 1, 33–42.

[B6] Beck A. T. Steer R. A. Brown G. K. (1996). Manual for the Beck Depression Inventory-II. San Antonio TX: Psychological Corporation.

[B8] Blood J. D. Wu J. Chaplin T. M. Hommer R. Vazquez L. Rutherford H. J.. (2015). The variable heart: high frequency and very low frequency correlates of depressive symptoms in children and adolescents. J. Affect. Disord. 186, 119–126. 10.1016/j.jad.2015.06.057 26233322 PMC4565756

[B9] Borkovec T. D. Robinson E. Pruzinsky T. DePree J. A. (1983). Preliminary exploration of worry: some characteristics and processes. Behav. Res. Ther. 21, 9–16. 10.1016/0005-7967(83)90121-3 6830571

[B10] Brosschot J. F. Gerin W. Thayer J. F. (2006). The perseverative cognition hypothesis: a review of worry, prolonged stress-related physiological activation and health. J. Psychosom. Res. 60, 113–124. 10.1016/j.jpsychores.2005.06.074 16439263

[B11] Brown T. A. Antony M. M. Barlow D. H. (1992). Psychometric properties of the Penn State Worry Questionnaire in a clinical anxiety disorders sample. Behav. Res. Ther. 30, 33–37. 10.1016/0005-7967(92)90093-v 1540110

[B12] Buzsáki G. Draguhn A. (2004). Neuronal oscillations in cortical networks. Science 304, 1926–1929. 10.1126/science.1099745 15218136

[B13] Castellanos F. X. Sonuga-Barke E. J. Scheres A. Di Martino A. Hyde C. Walters J. R. (2005). Varieties of attention-deficit/hyperactivity disorder-related intra-individual variability. Biol. Psychiatry 57, 1416–1423. 10.1016/j.biopsych.2004.12.005 15950016 PMC1236991

[B14] Chalmers J. A. Heathers J. A. Abbott M. J. Kemp A. H. Quintana D. S. (2016). Worry is associated with robust reductions in heart rate variability: a transdiagnostic study of anxiety psychopathology. BMC Psychol. 4:32. 10.1186/s40359-016-0138-z 27255891 PMC4891851

[B16] Conners C. K. (2000). Conners’ Continuous Performance Test II: Technical Guide. Toronto, ON: Multi-Health Systems.

[B17] Cropley M. Rydstedt L. W. Devereux J. J. Middleton B. (2015). The relationship between work-related rumination and evening and morning salivary cortisol secretion. Stress Health 31, 150–157. 10.1002/smi.2538 24166947

[B18] Davey G. C. (1993). A comparison of three worry questionnaires. Behav. Res. Ther. 31, 51–56. 10.1016/0005-7967(93)90042-s 8417728

[B19] Davydov D. M. Shapiro D. Cook I. A. Goldstein I. (2007). Baroreflex mechanisms in major depression. Prog. Neuropsychopharmacol. Biol. Psychiatry 31, 164–177. 10.1016/j.pnpbp.2006.08.015 17011098

[B20] De Luca M. Beckmann C. F. De Stefano N. Matthews P. M. Smith S. M. (2006). fMRI resting state networks define distinct modes of long-distance interactions in the human brain. Neuroimage 29, 1359–1367. 10.1016/j.neuroimage.2005.08.035 16260155

[B21] Di Martino A. Ghaffari M. Curchack J. Reiss P. Hyde C. Vannucci M.. (2008). Decomposing intra-subject variability in children with attention-deficit/hyperactivity disorder. Biol. Psychiatry 64, 607–614. 10.1016/j.biopsych.2008.03.008 18423424 PMC2707839

[B22] Dixon M. L. Fox K. C. R. Christoff K. (2014). A framework for understanding the relationship between externally and internally directed cognition. Neuropsychologia 62, 321–330. 10.1016/j.neuropsychologia.2014.05.024 24912071

[B23] Doucet G. Naveau M. Petit L. Delcroix N. Zago L. Crivello F.. (2011). Brain activity at rest: a multiscale hierarchical functional organization. J. Neurophysiol. 105, 2753–2763. 10.1152/jn.00895.2010 21430278

[B24] Doucet G. Naveau M. Petit L. Zago L. Crivello F. Jobard G.. (2012). Patterns of hemodynamic low-frequency oscillations in the brain are modulated by the nature of free thought during rest. Neuroimage 59, 3194–3200. 10.1016/j.neuroimage.2011.11.059 22155378

[B25] Fox E. Dutton K. Yates A. Georgiou G. A. Mouchlianitis E. (2015). Attentional control and suppressing negative thought intrusions in pathological worry. Clin. Psychol. Sci. 3, 593–606. 10.1177/2167702615575878 26504672 PMC4618297

[B31] Gazzellini S. Napolitano A. Bauleo G. Bisozzi E. Lispi M. L. Castelli E.. (2016). Time-frequency analyses of reaction times and theta/beta EEG ratio in pediatric patients with traumatic brain injury: a preliminary study. Dev. Neurorehabil. 14, 1–15. 10.1080/17518423.2016.1216470 27629793

[B33] Hayes S. Hirsch C. Mathews A. (2008). Restriction of working memory capacity during worry. J. Abnorm. Psychol. 117, 712–717. 10.1037/a0012908 18729625

[B34] Helps S. K. Broyd S. J. Bitsakou P. Sonuga-Barke E. J. (2011). Identifying a distinctive familial frequency band in reaction time fluctuations in ADHD. Neuropsychology 25, 711–719. 10.1037/a0024479 21728424

[B35] Helps S. James C. Debener S. Karl A. Sonuga-Barke E. J. (2008). Very low frequency EEG oscillations and the resting brain in young adults: a preliminary study of localisation, stability and association with symptoms of inattention. J. Neural Transm 115, 279–285. 10.1007/s00702-007-0825-2 17994187

[B36] Hirsch C. R. Mathews A. (2012). A cognitive model of pathological worry. Behav. Res. Ther. 50, 636–646. 10.1016/j.brat.2012.06.007 22863541 PMC3444754

[B37] Jennings J. R. Sheu L. K. Kuan D. C. Manuck S. B. Gianaros P. J. (2016). Resting state connectivity of the medial prefrontal cortex covaries with individual differences in high-frequency heart rate variability. Psychophysiology 53, 444–454. 10.1111/psyp.12586 26995634 PMC4800828

[B38] Johnson K. A. Kelly S. P. Bellgrove M. A. Barry E. Cox M. Gill M.. (2007a). Response variability in attention deficit hyperactivity disorder: evidence for neuropsychological heterogeneity. Neuropsychologia 45, 630–638. 10.1016/j.neuropsychologia.2006.03.034 17157885

[B39] Johnson K. A. Robertson I. H. Kelly S. P. Silk T. J. Barry E. Dáibhis A.. (2007b). Dissociation in performance of children with ADHD and high-functioning autism on a task of sustained attention. Neuropsychologia 45, 2234–2245. 10.1016/j.neuropsychologia.2007.02.019 17433378 PMC2000292

[B40] Knyazev G. G. Slobodskoj-Plusnin J. Y. Bocharov A. V. Pylkova L. V. (2011). The default mode network and EEG α oscillations: an independent component analysis. Brain Res. 1402, 67–79. 10.1016/j.brainres.2011.05.052 21683942

[B41] MacDonald S. W. Nyberg L. Bäckman L. (2006). Intra-individual variability in behavior: links to brain structure, neurotransmission and neuronal activity. Trends Neurosci. 29, 474–480. 10.1016/j.tins.2006.06.011 16820224

[B42] Makovac E. Meeten F. Watson D. R. Garfinkel S. N. Critchley H. D. Ottaviani C. (2015). Neurostructural abnormalities associated with axes of emotion dysregulation in generalized anxiety. Neuroimage Clin. 10, 172–181. 10.1016/j.nicl.2015.11.022 26759791 PMC4683456

[B43] McEvoy P. M. Watson H. Watkins E. R. Nathan P. (2013). The relationship between worry, rumination, and comorbidity: evidence for repetitive negative thinking as a transdiagnostic construct. J. Affect. Disord. 151, 313–320. 10.1016/j.jad.2013.06.014 23866301

[B44] Meloni F. Gana K. (2001). Wording effects in the Italian version of the Penn State Worry Questionnaire. Clin. Psychol. Psychother. 8, 282–287. 10.1002/cpp.294.abs

[B45] Meyer T. J. Miller M. L. Metzger R. L. Borkovec T. D. (1990). Development and validation of the Penn State Worry Questionnaire. Behav. Res. Ther. 28, 487–495. 10.1016/0005-7967(90)90135-6 2076086

[B46] Nolen-Hoeksema S. Morrow J. (1991). A prospective study of depression and posttraumatic stress symptoms after a natural disaster: the 1989 Loma Prieta earthquake. J. Pers. Soc. Psychol. 61, 115–121. 10.1037//0022-3514.61.1.115 1890582

[B47] Northoff G. Bermpohl F. (2004). Cortical midline structures and the self. Trends Cogn. Sci. 8, 102–107. 10.1016/j.tics.2004.01.004 15301749

[B48] Ottaviani C. Borlimi R. Brighetti G. Caselli G. Favaretto E. Giardini I.. (2014). Worry as an adaptive avoidance strategy in healthy controls but not in pathological worriers. Int. J. Psychophysiol. 93, 349–355. 10.1016/j.ijpsycho.2014.05.010 24873888

[B49] Ottaviani C. Medea B. Lonigro A. Tarvainen M. Couyoumdjian A. (2015). Cognitive rigidity is mirrored by autonomic inflexibility in daily life perseverative cognition. Biol. Psychol. 107, 24–30. 10.1016/j.biopsycho.2015.02.011 25749107

[B50] Ottaviani C. Shapiro D. Couyoumdjian A. (2013). Flexibility as the key for somatic health: from mind wandering to perseverative cognition. Biol. Psychol. 94, 38–43. 10.1016/j.biopsycho.2013.05.003 23680439

[B51] Ottaviani C. Thayer J. F. Verkuil B. Lonigro A. Medea B. Couyoumdjian A.. (2016a). Physiological concomitants of perseverative cognition: a systematic review and meta-analysis. Psychol. Bull. 142, 231–259. 10.1037/bul0000036 26689087

[B52] Ottaviani C. Watson D. R. Meeten F. Makovac E. Garfinkel S. N. Critchley H. D. (2016b). Neurobiological substrates of cognitive rigidity and autonomic inflexibility in generalized anxiety disorder. Biol. Psychol. 119, 31–34. 10.1016/j.biopsycho.2016.06.009 27345596

[B53] Penttonen M. Buzsáki G. (2003). Natural logarithmic relationship between brain oscillators. Thalamus Relat. Syst. 2, 145–152. 10.1017/s1472928803000074

[B55] Rapee R. M. (1993). The utilisation of working memory by worry. Behav. Res. Ther. 31, 617–620. 10.1016/0005-7967(93)90114-a 8347121

[B56] Ruscio A. M. (2002). Delimiting the boundaries of generalized anxiety disorder: differentiating high worriers with and without GAD. J. Anxiety Disord. 16, 377–400. 10.1016/s0887-6185(02)00130-5 12213034

[B57] Ruscio A. M. Borkovec T. D. Ruscio J. (2001). A taxometric investigation of the latent structure of worry. J. Abnorm. Psychol. 110, 413–422. 10.1037//0021-843x.110.3.413 11502084

[B58] Salzer S. Stiller C. Tacke-Pook A. Jacobi C. Leibing E. (2009). Screening for generalized anxiety disorder in inpatient psychosomatic rehabilitation: pathological worry and the impact of depressive symptoms. Psychosoc. Med. 6:Doc02. 10.3205/psm000058 19742048 PMC2736478

[B59] Schneider W. Eschman A. Zuccolotto A. (2002). E-Prime User’s Guide. Pittsburgh, PA: Psychology Software Tools.

[B60] Sonuga-Barke E. J. Castellanos F. X. (2007). Spontaneous attentional fluctuations in impaired states and pathological conditions: a neurobiological hypothesis. Neurosci. Biobehav. Rev. 31, 977–986. 10.1016/j.neubiorev.2007.02.005 17445893

[B61] Spielberger C. D. Gorsuch R. L. Lushene R. E. (1970). STAI Manual. Palo Alto, CA: Consulting Psychologists Press.

[B62] Tarvainen M. P. Niskanen J. P. Lipponen J. A. Ranta-Aho P. O. Karjalainen P. A. (2014). Kubios HRV—heart rate variability analysis software. Comput. Methods Programs Biomed. 113, 210–220. 10.1016/j.cmpb.2013.07.024 24054542

[B63] Task Force of the European Society of Cardiology and the North American Society of Pacing and Electrophysiology (1996). Heart rate variability: standards of measurement, physiological interpretation, and clinical use. Circulation 93, 1043–1065. 10.1161/01.cir.93.5.1043 8598068

[B64] Thayer J. F. Åhs F. Fredrikson M. Sollers J. J. III Wager T. D. (2012). A meta-analysis of heart rate variability and neuroimaging studies: implications for heart rate variability as a marker of stress and health. Neurosci. Biobehav. Rev. 36, 747–756. 10.1016/j.neubiorev.2011.11.009 22178086

[B660] Vanhatalo S. Palva J. M. Holmes M. D. Miller J. W. Voipio J. Kaila K. (2004). Infraslow oscillations modulate excitability and interictal epileptic activity in the human cortex during sleep. Proc. Natl. Acad. Sci. U S A 101, 5053–5057. 10.1073/pnas.0305375101 15044698 PMC387372

[B65] Van Rijsoort S. Emmelkamp P. Vervaeke G. (1999). The Penn State Worry Questionnaire and the worry domains questionnaire: structure, reliability and validity. Clin. Psychol. Psychother. 6, 297–307. 10.1002/(SICI)1099-0879(199910)6:4<297::AID-CPP206>3.0.CO;2-E

[B66] Weissman D. H. Roberts K. C. Visscher K. M. Woldorff M. G. (2006). The neural bases of momentary lapses in attention. Nat. Neurosci. 9, 971–978. 10.1038/nn1727 16767087

